# Translation and validation study of the Persian version of the Arthritis Impact Measurement Scales 2 (AIMS2) in patients with osteoarthritis of the knee

**DOI:** 10.1186/1471-2474-10-95

**Published:** 2009-08-01

**Authors:** Sayed Javad Mousavi, Mohamad Parnianpour, Ahmad Reza Askary-Ashtiani, Mohamad Reza Hadian, Abdolrahman Rostamian, Ali Montazeri

**Affiliations:** 1 Department of Physical Therapy, Faculty of Rehabilitation Sciences, Tehran University of Medical Sciences, Tehran, Iran; 2 Department of Mechanical Engineering, Sharif University of Technology, Tehran, Iran; 3 Information and Industrial Engineering, Han Yang University, Seoul, Korea; 4 Department of Physical Therapy, Faculty of Paramedical Sciences, Zahedan University of Medical Sciences, Zahedan, Iran; 5 Sport Medicine Research Center (SMRC), Tehran University of Medical Sciences, Tehran, Iran; 6 Department of Rheumatology, Faculty of Medicine, Tehran University of Medical Sciences, Tehran, Iran; 7 Department of Mental Health, Iranian Institute for Health Sciences Research, ACECR, Tehran, Iran

## Abstract

**Background:**

The Arthritis Impact Measurement Scales 2 (AIMS2) has not been translated and validated for Persian-speaking patients with osteoarthritis of the knee. This was to provide a validated instrument to measure functional disability and health-related quality of life in patients with osteoarthritis of the knee in Iran. The aim of this study was to culturally adapt and validate the AIMS2 for Persian-speaking patients with osteoarthritis of the knee in Iran.

**Methods:**

A consecutive sample of patients with knee osteoarthritis were asked to complete the AIMS2, the Short Form Health Survey (SF-36) and four visual analog scales for pain, joint stiffness, patient's and physician's global assessment. Internal consistency and convergent validity were applied to examine psychometric properties of the AIMS2. In addition, 30 randomly selected patients were asked to complete the questionnaire two days later for the second time for test-retest reliability. Finally factor structure of the Persian AIMS2 was performed using the principal component factor analysis.

**Results:**

In all 230 patients were entered into the study. The mean (SD) age of the participants was 56.9 (8.7) years and the mean (SD) duration of disease was 7.2 (3.5) years. Cronbach's alpha coefficient and intraclass correlation coefficient (ICC) for the Persian AIMS2 scales ranged from 0.74 to 0.92 and 0.85 to 0.96, respectively. The correlation between most of the Persian AIMS2 scales and the physical and mental summary scores of the SF-36 and the visual analogue scales for pain, joint stiffness, patient's and physician's global assessment were statistically significant indicating a good convergent validity (p < 0.05). The results obtained from factor analysis indicated three latent factors that jointly accounted for 67.5% of the total variance.

**Conclusion:**

The results showed that the Persian AIMS2 had reasonably good internal consistency, test-retest reliability, and convergent validity in patients with osteoarthritis of the knee. It is simple and easy to use and now can be applied in the future studies in Iran. However, its sensitivity to change needs still to be studied.

## Background

Osteoarthritis (OA) is a major medical, social, and economic problem in both developed and developing countries [1-3]. It often affects a variety of physical and psychosocial health domains, from fairly basic self-care activities to advance and complex social, work, and leisure activities and eventually has a profound impact on health related quality of life (HRQOL) [2,4,5]. Osteoarthritis of the knee is the most prevalent degenerative joint disease and a major cause of disability, particularly in the aging population [6,7]. Cross-sectional and prospective studies found that HRQOL is negatively affected in patients with OA of the knee [2,3,8,9]. The aim of contemporary treatment in most of the rheumatoid and orthopaedic conditions, including OA of the knee, is often to improve the functional ability and quality of life of the patient; hence health status should be considered as an important outcome in clinical trials investigating OA of the knee [10,11].

The Arthritis Impact Measurement Scales 2 (AIMS2) is a shorter version of the AIMS and has been developed for the purpose of assessing the impact of arthritis on patient's quality of life [12,13]. This short version has been shown to be more comprehensive, and acceptable to patients than AIMS while it takes a shorter time to be completed [13]. Several studies of patients with rheumatoid arthritis proved that it is as reliable and valid as the longer version [13-15]. This questionnaire has been translated into many different languages [14-20]. The psychometric properties of the Italian [17], Chinese [18], Turkish [19], and German [20] versions of the AIMS2 have been evaluated in patients with OA of the knee and/or hip.

There is an increasing need for internationally standardized instruments to measure health status in patients with OA of the knee in a manner that allows comparison across cultures and countries [21,22]. Osteoarthritis of the knee is the most common disease among adults in Iran, with reported 7-days period prevalence of symptomatic OA as high as 15.3% in urban [23] and 19.3% in rural areas [24]. Given the population of Iran (over 70 million) and high prevalence of OA of the knee in the country, the AIMS2, as an internationally recognized instrument for patients with osteoarthritis, was not yet validated in Iran. This study aimed to translate and culturally adapt the AIMS2 for use with Persian-speaking patients with OA of the knee in Iran, and to investigate its psychometric properties.

## Methods

### Translation and cultural adaptation

Forward-backward procedure was applied to translate the English version of the AIMS2 into Persian (the Iranian language). Two independent professional translators produced two forward translations. Both translators were instructed to aim for conceptual rather than literal translation. Together with one of the authors, the translators compared their translations and produced a single provisional version of the questionnaire. Totally blind to the original version, two other professional translators translated the provisional Persian questionnaire back into the English language. The two translators were not aware of the questionnaire. Finally, an expert committee consisted of the translators, the researchers, two rheumatologists, one orthopedics surgeon and one specialist in psychometric reviewed all the translation and cultural adaptation processes. They also evaluated the final English backward version with the original questionnaire. Consensus in terms of semantic, idiomatic, experiential, and conceptual equivalence was reached and a pre-final version of the questionnaire (the Persian AIMS2) was provided. In general the original scales were remained unchanged except some minor modifications in items 7, 9 and 33. In items 7 and 9, 'walking several blocks' and 'walking one block' have been changed to 'walking several alleys' and 'walking one alley', respectively to refer to a similar distance in Persian language. In item 33, 'church' was changed to 'religious meetings' to imply comparable meaning.

### The AIMS2

The AIMS2 consists of 78 items and the first 57 items are broken to 12 scales and measuring: mobility level (5 items), walking and bending (5 items), hand and finger function (5 items), arm function (5 items), self care (4 items), household tasks (4 items), social activities (5 items), support from family and friends (4 items), arthritis pain (5 items), work (5 items), level of tension (5 items) and mood (5 items). Scores on each scale range from 0 to 10 with lower scores indicating better quality of life [12]. Usually these scales refer to the shorter version of AIMS2 that was used in this study and should not be confused with the 'Short Form' version of the AIMS2 known as AIMS2-SF.

However, the remaining 21 items of the questionnaire are related to satisfaction with each area of health (1 item), arthritis impact on each area of health (1 item), three areas most like to see improvement (1 item), current and future health (5 items), overall arthritis impact (3), medication (1 item), co-morbidity (3 item), socio-economic status (6 items).

### Additional measures

#### The SF-36

The SF-36 is a general health questionnaire that consists of 36 items, summarized into eight subscales. The eight subscales are summarized into physical component scores (PCS) and mental component score (MCS). The PCS and MCS provide greater precision, and reduce the number of statistical comparisons needed [25]. The psychometric properties of the Iranian version of SF-36 are well documented [26].

#### Clinical Measures

The patient's perception of pain, joint stiffness, and global severity assessment level was measured on a 100 mm visual analogue scale (VAS) ranging from zero (representing 'no pain', 'no stiffness', 'no problem') to 100 (representing 'maximum imaginable pain', 'extreme stiffness' or ' extremely severe)'. Similarly, the physician's global assessment of disease severity was recorded using a 100 mm VAS anchored by word descriptors at each end (0 = 'asymptomatic', 100 = 'extremely severe').

#### Patients

A consecutive sample of 230 patients with symptomatic OA of the knee for at least one year duration who were referred to 6 rheumatology and physical therapy clinics in Tehran, Iran, were entered into the study over a period of 11 months. All patients fulfilled clinical and radiological criteria for OA of the knee according to the American College of Rheumatology (ACR) [27]. A rheumatologist classified severity of radiological severity of OA of the knee according to the Kellgren-Lawrence standard criteria. This is a four-grade scale with higher grade indicating more severity [28]. In order to carry out the test-retest reliability a total of 30 patients, randomly selected from the original group, completed the Persian AIMS2 again 2 days later, in the same manner as the first one. Patients with recent lower extremity fractures and operation, inflammatory arthritis (e.g., rheumatoid arthritis), systematic inflammatory disease, and relevant comorbidities such as cancer, infectious disease, heart attack or clinically recognizable cognitive impairment were excluded from the study.

### Statistical analysis

#### Distribution of data

Kolmogorov-Smirnov test was used to assess distribution of the Persian AIMS2, MCS, PCS, and the VAS scores. The interval measurements were normally distributed, and therefore several parametric tests were employed to analyze data. The critical values for significance were set at p < 0.05.

#### Reliability

Reliability of measurement refers to the extent, to which the measured variance in a score reflects the true score, rather than random error; that is, the extent to which measures give consistent or accurate results [29]. Two common forms of reliability are internal consistency method and test-retest reliability. The internal consistency of a scale relates to its homogeneity. The higher the coefficient value, the higher the reliability and the lower the standard error of measurement. The internal consistency was assessed with the Cronbach's alpha coefficient that ranges from zero to 1 and values equal or greater than 0.70 indicate adequate internal consistency for a scale [29]. Test-retest reliability measures stability over time, by administering the same test to the same subjects at two points in time [29]. In this study, 30 randomly selected patients completed the Persian AIMS2 twice with a time interval of 2 days. Test-retest reliability was examined using Intraclass Correlation Coefficient (ICC). Values of ICC vary from zero (totally unreliable) to 1 (perfectly reliable) and values above 0.80 were considered as evidence of excellent reliability [29].

#### Convergent validity

Convergent validity examines the extent to which a particular measure relates to other measures that is believed to be assessing the same construct [30]. In the absence of a true 'gold standard" against which to assess criterion validity of the Persian AIMS2, we compared this questionnaire with commonly used external measures likely to reflect the impact of knee OA. Thus, correlation between the Persian AIMS2 and the physical and mental component summary scores of the SF-36, and clinical measures (i.e. patient's pain, joint stiffness, global assessment, and physician's global assessment of disease severity) was measured. It was expected that physical constructs of the AIMS2 would be correlated with physical, and mental constructs of the AIMS2 would be correlated with mental components of the SF-36. The Pearson's correlation coefficient was used to test convergent validity of the Persian AIMS2. Correlation values of 0.40 or above were considered satisfactory (r ≥ 0.81–1.0 as excellent, 0.61–0.80 very good, 0.41–0.60 good, 0.21–0.40 fair, and 0–0.20 poor) [29,31].

#### Factor analysis

Finally factor analysis was applied to determine the dimensionality of the items of a questionnaire that is, to determine whether they formed only one overall dimension or more than one [31]. Factor structure of the Persian AIMS2 was performed using the principal component factor analysis with varimax rotation.

#### Ethics

The Ethics Committee of the Tehran University of Medical Sciences approved the study. All patients gave informed consent.

## Results

Table 1 summarizes the demographic and clinical characteristics of the study population as well as descriptive statistics of the distribution for the scales. The mean (SD) age of the participants was 56.9 (8.7) years ranging from 36 to 76. Sixty-eight percents of the respondents were female. The mean duration of disease was 7.2 ± 3.5 years. At the time of enrollment, 80% of the subjects were working (considering household work also as work for women), 12% were retired, and 8% had stopped working because of their knee pain. All subjects were affected by OA of the knee with a radiological severity mostly of the second and third degrees on Kellgren-Lawrence's scale (49.2% and 35.5%, respectively). The mean time to complete the Persian AIMS2 was approximately 23 minutes. In general most participants found the Persian AIMS2 easy to complete and relevant to their current lower extremity problem. Most patients (95%) completed the questionnaire without any missing data.

**Table 1:** Demographic and clinical characteristics of the study population and distribution of scores for each questionnaire (n = 230)

		**Mean**	**SD**
**Age**	Years	56.9	8.7

**Disease duration**	Years	7.2	3.5

**SF-36 (0–100)**			

	PCS	33.4	8.2

	MCS	44.2	9.9

**VAS (0–100)**			

	Pain	55.2	22.1

	Stiffness	48.4	16.5

	Patient's assessment	58.6	12.9

	Physician's assessment	49.7	15.4

**Kellgren-Lawrence score (%)**		2.7	0.79

	Grade I	28	8.5

	Grade II	163	49.4

	Grade III	117	35.5

	Grade IV	22	6.6

**AIMS2 Scales (0–10)**			

	Mobility level	3.02	2.1

	Walking and bending	6.0	2.34

	Hand and finger function	1.05	1.1

	Arm function	1.41	1.5

	Self-care	1.2	1.4

	House holding tasks	1.98	2.1

	Social activities tasks	4.93	2.4

	Support from family and friends	2.1	1.5

	Arthritis pain	5.89	2.5

	Work	3.98	1.8

	Level of tension	5.1	1.9

	Mood	3.4	1.4

AIMS2: Arthritis Measurement Scales 2; SF-36: Short Form Health Survey; PCS: Physical Component Summary; MCS: Mental Component Summary, VAS: Visual Analogue Scale.

### Reliability

Table 2 shows the main results of scale reliability analyses. Cronbach's coefficient ranged from 0.74 for arm function to 0.92 for work. Intraclass correlation coefficient value for the Persian AIMS2 ranged from 0.85 for social activities to 0.96 for mobility level, which indicates excellent test-retest reliability for the AIMS2 scales.

**Table 2:** Internal consistency and test-retest reliability of the Persian AIMS2

	**Internal consistency (Cronbach's alpha)**	**Test-retest reliability (ICC)**
Mobility level	0.83	0.94

Walking and bending	0.81	0.92

Hand and finger function	0.84	0.90

Arm function	0.79	0.89

Self-care	0.89	0.93

Household tasks	0.88	0.91

Social activities	0.84	0.92

Support from family and friends	0.78	0.85

Arthritis pain	0.83	0.90

Work	0.81	0.94

Level of tension	0.84	0.89

Mood	0.79	0.94

AIMS2: Arthritis Measurement Scales 2; ICC: Intraclass Correlation Coefficient.

### Convergent validity

Table 3 summarizes the statistical analyses of correlation between the Persian AIMS2 and the external measures, using Pearson's correlation coefficient. Good significant correlations were found between the PCS and most of the Persian AIMS2 scales, except for support from family and friends, level of tension and mood scales as expected. The PCS had the highest correlation with mobility level, walking and bending and arthritis pain and lowest correlation with support from family and friends lending support to our hypothesis. The MCS were significantly correlated with most of the Persian AIMS2 scales. The highest correlation was observed between the MCS and tension and mood scale scores as predicted.

**Table 3:** The Pearson correlation coefficients of the Persian AIMS2 and the SF-36 component scores and clinical measures (n = 230)

	**PCS**	**MCS**	**Pain**	**Stiffness**	**Patient's assessment**	**Physician's assessment**
Mobility level	-0.50**	-0.18*	0.44**	0.40**	0.41*	0.45**

Walking and bending	-0.67**	-0.22*	0.55**	0.45**	0.50*	0.58**

Hand and finger function	-0.34*	-0.10*	0.13*	0.11*	0.20*	0.21*

Arm function	-0.36*	-0.05	0.22*	0.19*	0.31*	0.32*

Self-care	-0.18*	-0.10	0.30*	0.20*	0.23*	0.22*

Household tasks	-0.27*	-0.15*	0.18*	0.17*	0.14*	0.25*

Social activities	-0.40**	-0.45**	0.10	0.005	-0.01	0.17*

Support from family and friends	-0.05	-0.38*	0.09	0.1	0.03	0.08

Arthritis pain	-0.71**	-0.29*	0.57**	0.49**	0.47**	0.50*

Work	-0.58*	-0.31*	0.49**	0.51**	0.55**	0.48**

Level of tension	-0.29*	-0.41*	0.29*	0.27*	0.21*	0.23*

Mood	-0.39*	-0.49**	0.27*	0.32*	0.22*	0.37**

* P < 0.05, ** P < 0.01

AIMS2: Arthritis Measurement Scales 2; SF-36: Short Form Health Survey; PCS: Physical Component Summary; MCS: Mental Component Summary, VAS: Visual Analogue Scale.

Moderate to high correlations were observed between mobility level, walking and bending, work, and level of tension scales and VAS measures for pain, joint stiffness, patient's and physician's global assessment. The highest correlations were found between arthritis pain scale and the VAS for pain and joint stiffness. The scores for hand and finger function, arm function, household tasks and mood scales were moderately correlated with most measures. The social activities and support from family and friends scales were not significantly correlated with clinical measures.

### Factor analysis

A factor analysis with varimax rotation was performed and 3 latent factors extracted with eigenvalues greater than 1 that jointly accounted for 67.5% of the total variance. The lower-limb (mobility level and walking and bending) and upper-limb (hand and finger function, arm function) functioning scales together with the self care, household tasks, arthritis pain and work scales formed the first factor, which explained 47% of the total variance measured. The psychological scales (level of tension and mood) were loaded on the second factor, which explained 11.1% of the variance. The third factor (9.4% of the variance) was determined by the social dimension, consisting of the social activities and support from family and friends scales (Table 4).

**Table 4:** The factor structure of the Persian AIMS2 obtained from principal component analysis*

	**Factor 1**	**Factor 2**	**Factor 3**
	**Physical**	**Psychological**	**Social**

**AIMS2 scales**		-	-

Mobility level	0.72	-	-

Walking and bending	0.65	-	-

Hand and finger function	0.59	-	-

Arm function	0.54	-	-

Self-care	0.74	-	-

Household tasks	0.75	-	-

Social activities		-	0.60

Support from family and friends		-	0.57

Arthritis pain	0.65	-	-

Work	0.53	-	-

Level of tension	-	0.76	-

Mood	-	0.67	-

Variance contributed by each factor**	47	11.1	9.4

* Factor loadings greater than 0.40 were considered satisfactory.

** Total variance explained: 67.5%.

## Discussion

The results of present study have shown satisfactory internal consistency, test-retest reliability, and convergent validity for the Persian AIMS2 in patients with osteoarthritis of the knee. The questionnaire covers most important areas of health status that are influenced by knee OA. With the growing number of multinational and multi-center studies, the need to culturally adapt health status measures for use in other than the original source language has grown rapidly [32]. The presence of an internationally validated instrument for knee OA would allow direct comparison across countries and centers.

Our findings suggested satisfactory internal consistency for the Persian AIMS2. The Cronbach's alpha of the Persian AIMS2 ranged from 0.74 to 0.91 that is comparable to results previously reported for Italian (0.72–0.92), Turkish (0.72–0.90), and German (0.73–0.88) versions of the AIMS2 [17,19,20]. In addition, the Persian AIMS2 showed excellent test-retest reliability, with ICC ranging from 0.83 to 0.96. Similar ICC values have been reported in Italian (0.70–90), Turkish (0.75–0.98), and German studies (0.87–0.95) [17,19,20].

The Persian AIMS2 showed significant good convergent validity, as assessed with the physical and mental component scores of the Persian SF-36 and clinical measures. Satisfactory significant correlations were found between the PCS score and most of the Persian AIMS2 scales, especially mobility level, walking and bending, and arthritis pain. Correlation between support from family and friends and the PCS was not significant. Fair to good correlations were observed between MCS and most of the AIMS2 scales. The highest correlation was found between the MCS and mood, social activities and level of tension. No significant correlation was found between the MCS and arm function and self care. In a Chinese validation study the World Health Organization Quality of Life questionnaire (WHOQOL-BREF) was used to test convergent validity of the AIMS2. In their study significant correlation were reported between physical and psychological dimensions of the WHOQOL-BREF and the Chinese AIMS2 [18]. However, our findings demonstrated that the Persian version of the AIMS2 is a valid instrument to measure physical and psychosocial of HRQOL in patients with OA of the knee.

Fair to good correlations were observed between most of the Persian AIMS2 scales and the subjective clinical measures of pain, joint stiffness and global assessment of disease severity. All or some of these clinical measures were used in previous studies to test convergent validity of the Italian [17], Chinese [18], Turkish [19], and German [20], version of the AIMS2. Stronger correlation between mobility level and walking and bending scales and all clinical measures were found in the present study relative to other validation studies. Similar to German validation [20], the correlation between social activity scale and clinical measures was significant.

A three-factor solution was extracted from the factor analysis of the Persian AIMS2 that jointly accounted for the 67.5% of the total variance. In fact the result was satisfactory and consistent with the theoretical basis for the original conceptual model of the instrument. A German study also confirmed the postulated three-factor structure for the AIMS2 with a physical, physiological and social dimension, explaining 48.5%, 13.9% and 6.8% of the variation, respectively [20]. Similarly an Italian study indicated that the factor analysis provided a three-factor health status model explaining 63.5% of the variance observed [17].

This study has several limitations. Perhaps the main concern is that this study did not provide evidence for responsiveness to change or other psychometric tests (e.g. discriminant validity). Secondly, the statistical analysis was limited. For instance, to indicate the factor structure of the instrument it would be interesting to carry out confirmatory factor analysis instead of exploratory factor analysis. The future studies could focus on other psychometric properties of the questionnaire and also on different applications of the AIMS2 among Iranian patients.

## Conclusion

The present study presents initial step in evaluating psychometric properties of a well-known instrument to measure the impact of arthritis on Persian-speaking patients with osteoarthritis of the knee in Iran. Since health related quality of life was rarely assessed as primary end-point in studies of osteoarthritis of the knee in Iran, indeed the Persian AIMS2 might possibly provide both clinicians and patients with numerous advantages as an important outcome measure in the future studies. However, its sensitivity to change needs still to be studied.

## Competing interests

The authors declare that they have no competing interests.

## Authors' contributions

All authors were involved in data collection, analysis, interpretation of results, and manuscript preparation. In addition, SJM, MP, and AM designed the study and prepared the first draft of the paper. AM provided the final manuscript. All authors read and approved the final manuscript.

## Pre-publication history

The pre-publication history for this paper can be accessed here:


